# Effect of stimulation parameters on entrainment

**DOI:** 10.1016/j.hroo.2022.09.012

**Published:** 2022-09-21

**Authors:** Sen Lei, Feng-Peng Jia, Quan He, Ling-Yun Gao, Su-Cin Luo

**Affiliations:** Department of Cardiovascular Medicine, The First Affiliated Hospital of Chongqing Medical University, Chongqing, China

**Keywords:** Atrial flutter, Entrainment, Postpacing interval, Stimulation parameter, Virtual electrode

**Key Findings**

▪ Increasing stimulation intensity may shorten the postpacing interval (PPI).
▪ Pacing rate may affect PPI.
▪ Number of stimuli does not affect PPI.

The postpacing interval (PPI) may be longer (>30 ms) or even shorter (<0 ms) than the tachycardia cycle length (TCL) when entrainment pacing is performed within the isthmus of the circuit.^1^^,^^2^ In this study, we aimed to evaluate whether stimulation parameters can affect the PPI in typical (cavotricuspid isthmus–dependent) atrial flutter (AFL).

Twenty-one patients with typical AFL were included in the study. Each patient provided written informed consent to the study protocol, which was approved by the Institutional Ethics Committee (Research No. 2020FYYX032). The research reported in this paper adhered to Helsinki Declaration.

After confirmation of a typical AFL, entrainment pacing with different parameters was performed. Pacing trains were performed with different numbers of pacing stimuli (16, 24, and 32 basic S1S1 stimulations), different stimulation intensities (5, 8, and 10 mA), and different pacing rates (10 ms less than TCL, 20 ms less than TCL, 30 ms less than TCL).

Continuous variables (PPI–TCL) were compared using the paired *t* test or Wilcoxon signed rank test. *P* <.05 was considered significant. All analyses were performed using SPSS Version 17.0 (SPSS Inc., Chicago, IL).

**Figure 1:**
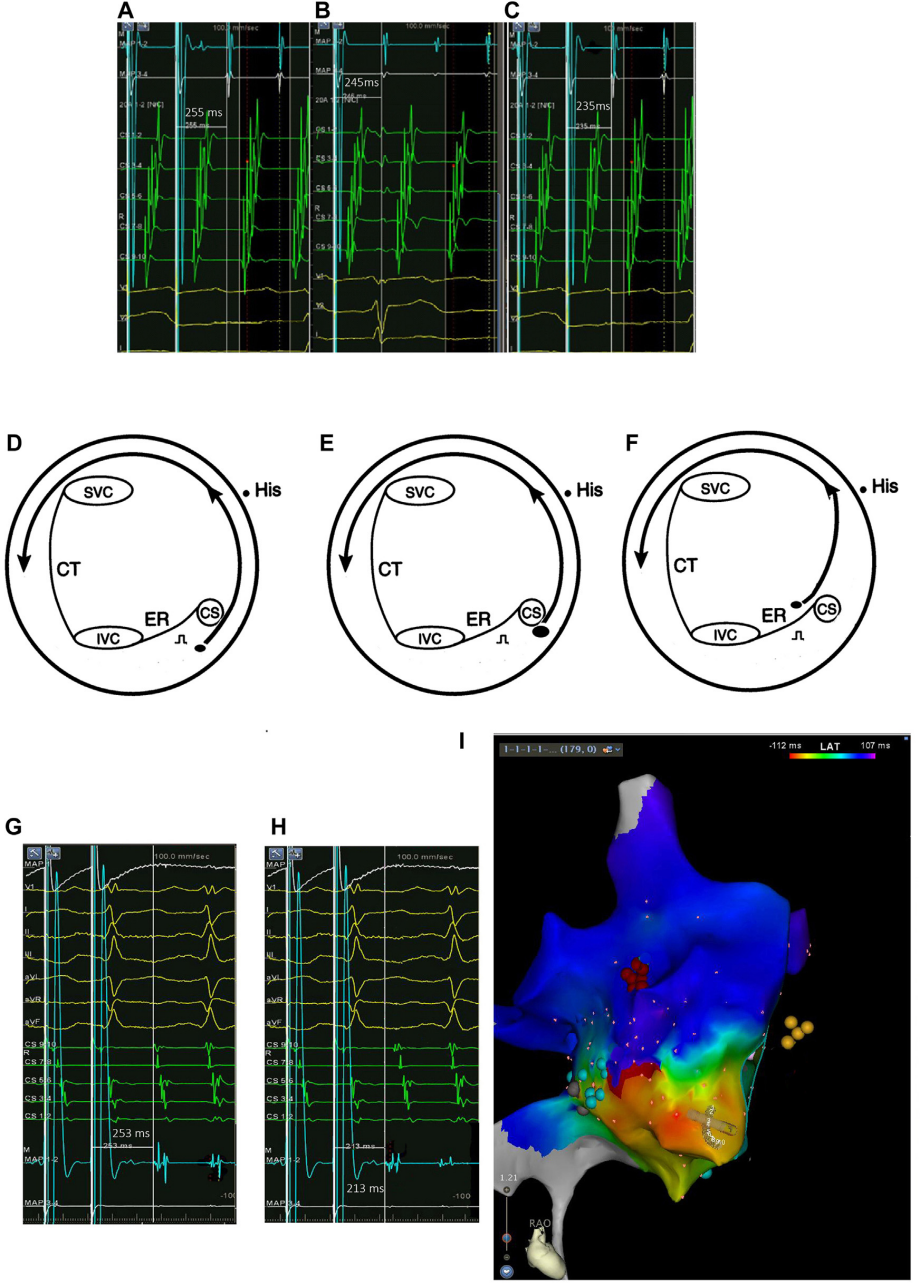
**A:** Entrainment pacing in the isthmus at a stimulus intensity of 5 mA with a postpacing interval (PPI) of 255 ms. The tachycardia cycle length (TCL) was 255 ms, and pacing rate was 245 ms. **B:** Entrainment pacing in the isthmus at a stimulus intensity of 8 mA with a PPI of 246 ms. **C:** Entrainment pacing in the isthmus at a stimulus intensity of 10 mA with a PPI of 235 ms. **D:** Pacing at lower intensity. The virtual electrode *(oval)* is next to the pacing site. **E:** Pacing at higher intensity. The virtual electrode is farther away. **F:** If pacing near the scar or the area of conduction block (eg, eustachian ridge [ER]), the pacing virtual electrode may capture one or the other side of the barrier side. The circuit may be changed and the PPI changed significantly. **G:** Pacing at lower intensity. PPI was 253 ms, TCL was 220 ms, and pacing rate was 210 ms. **H:** Pacing at higher intensity. PPI was 213 ms. Note the double potentials recorded at the pacing electrode. **I:** Three-dimensional mapping showing typical atrial flutter. *Yellow tags* represent the His bundle; *red tags* represent ablation sites; *black tags* represent scars; and *blue tags* represent double potentials. CS = coronary sinus; CT = crista terminalis; IVC = inferior vena cava; SVC = superior vena cava.

There was no significant difference in PPI–CL between 16 and 24 basic S1S1 stimulations (12.0 ± 11.1 ms vs 12.2 ± 11.0 ms, respectively; *P* = .55) and between 24 and 32 basic S1S1 stimulations (12.2 ± 11.0 ms vs 11.4 ± 10.4 ms, respectively; *P* = .05). This finding indicates that the number of pacing stimuli does not affect PPI. There was a significant difference in PPI–CL between stimulation intensities of 5 and 8 mA (11.4 ± 10.5 ms vs 7.1 ± 8.7 ms; *P* = .01) and between stimulation intensities of 8 and 10 mA (7.1 ± 8.7 ms vs 3.20 ± 7.6 ms; *P* = .01). An example is shown in Figures 1A to 1C. Figures 1D and 1E show the possible mechanisms. When stimulation intensity increases, more of the far-field tissue may be captured because of the virtual electrode and the downstream wavefront forming farther from the pacing site and then returning to the pacing site earlier. As a result, PPI is shortened. Despite the existence of variability in PPI,^3^ we can clearly see this tendency. Although the difference observed when the entrainment parameters are changed can consist of only a few milliseconds, it could lead to misclassification of an entrained site when the PPI–TCL interval is close to the threshold of 30 ms. The possible mechanism and an example is shown in Figures 1F to 1I.

There was a significant difference in PPI-CL between pacing with 10 ms less than TCL and 20 ms less than TCL (9.3 ± 5.9 ms vs 10.6 ±6.2 ms; *P* <.001), and between pacing with 20 ms less than TCL and 30 ms less than TCL (10.6 ± 6.2 ms vs 9.2 ±7.3 ms, respectively; *P* = .04). Faster pacing may lead to decremental conduction or concealed conduction, which may affect PPI.^4^

In conclusion, PPI is related to stimulation intensity and rate. Increasing stimulation intensity may shorten PPI, and the fast pacing rate also may affect PPI.
